# Adverse Life Experiences and Brain Function: A Meta-Analysis of Functional Magnetic Resonance Imaging Findings

**DOI:** 10.1001/jamanetworkopen.2023.40018

**Published:** 2023-11-01

**Authors:** Niki Hosseini-Kamkar, Mahdieh Varvani Farahani, Maja Nikolic, Kaycee Stewart, Samantha Goldsmith, Mahdie Soltaninejad, Reza Rajabli, Cassandra Lowe, Andrew A. Nicholson, J. Bruce Morton, Marco Leyton

**Affiliations:** 1 Now with: Atlas Institute for Veterans and Families, Royal Ottawa Hospital, Ottawa, Ontario, Canada; 2 Department of Psychiatry, McGill University, Montreal, Quebec, Canada; 3 Department of Medical Biophysics, Western University, London, Ontario, Canada; 4 Department of Psychology, Western University, London, Ontario, Canada; 5 McConnell Brain Imaging Centre, Montreal Neurological Institute, McGill University, Montreal, Quebec, Canada; 6 Department of Psychology, University of Exeter, Exeter, United Kingdom; 7 Department of Psychology, University of Ottawa, Ottawa, Ontario, Canada

## Abstract

**Importance:**

Adverse life experiences have been proposed to contribute to diverse mental health problems through an association with corticolimbic functioning. Despite compelling evidence from animal models, findings from studies in humans have been mixed; activation likelihood estimation (ALE) meta-analyses have failed to identify a consistent association of adverse events with brain function.

**Objective:**

To investigate the association of adversity exposure with altered brain reactivity using multilevel kernel density analyses (MKDA), a meta-analytic approach considered more robust than ALE to small sample sizes and methodological differences between studies.

**Data Sources:**

Searches were conducted using PsycInfo, Medline, EMBASE, and Web of Science from inception through May 4, 2022. The following search term combinations were used for each database: *trauma*, *posttraumatic stress disorder* (*PTSD*), *abuse*, *maltreatment*, *poverty*, *adversity*, or *stress*; and *functional magnetic resonance imaging* (*fMRI*) or *neuroimaging;* and *emotion*, *emotion regulation*, *memory*, *memory processing*, *inhibitory control*, *executive functioning*, *reward*, or *reward processing*.

**Study Selection:**

Task-based fMRI studies within 4 domains (emotion processing, memory processing, inhibitory control, and reward processing) that included a measure of adverse life experiences and whole-brain coordinate results reported in Talairach or Montreal Neurological Institute space were included. Conference abstracts, books, reviews, meta-analyses, opinions, animal studies, articles not in English, and studies with fewer than 5 participants were excluded.

**Data Extraction and Synthesis:**

Using the Preferred Reporting Items for Systematic Reviews and Meta-Analyses reporting guideline, 2 independent reviewers assessed abstracts and full-text articles for entry criteria. A third reviewer resolved conflicts and errors in data extraction. Data were pooled using a random-effects model and data analysis occurred from August to November 2022.

**Main Outcomes and Measures:**

Peak activation x-axis (left-right), y-axis (posterior-anterior), and z-axis (inferior-superior) coordinates were extracted from all studies and submitted to MKDA meta-analyses.

**Results:**

A total of 83 fMRI studies were included in the meta-analysis, yielding a combined sample of 5242 participants and 801 coordinates. Adversity exposure was associated with higher amygdala reactivity (familywise error rate corrected at *P* < .001; x-axis = 22; y-axis = −4; z-axis = −17) and lower prefrontal cortical reactivity (familywise error rate corrected at *P* < .001; x-axis = 10; y-axis = 60; z-axis = 10) across a range of task domains. These altered responses were only observed in studies that used adult participants and were clearest among those who had been exposed to severe threat and trauma.

**Conclusions and Relevance:**

In this meta-analysis of fMRI studies of adversity exposure and brain function, prior adversity exposure was associated with altered adult brain reactivity to diverse challenges. These results might better identify how adversity diminishes the ability to cope with later stressors and produces enduring susceptibility to mental health problems.

## Key Points

**Question:**

Is prior adversity exposure associated with changes to brain reactivity later in life?

**Findings:**

This multilevel kernel density meta-analysis of 83 neuroimaging studies found that prior adversity was associated with greater amygdala reactivity and diminished prefrontal cortex reactivity across a wide range of task domains.

**Meaning:**

These results might better identify how adverse life experiences diminish the ability to cope with later stressors and heighten susceptibility to mental illnesses.

## Teaser

This meta-analysis investigates the association of adverse life experiences and altered brain reactivity measured with functional magnetic resonance imaging (MRI).

## Introduction

Adverse life experiences have been associated with an increase in risk for mental illness, putatively by inducing long-lasting changes to brain function.^1,2,3,4,5,6,7^ Of particular interest is stress-induced neuroplasticity within regions that appraise potential threats and initiate behavioral and physiologic coping responses^8,9,10,11^ such as the prefrontal cortex (PFC), amygdala, hippocampus, and parts of the salience network.^12,13,14,15,16,17,18,19,20^ Studies in laboratory animals^8,9,10,11^ support this hypothesis, but evidence in humans is mixed, with adversity being associated with increased,^21,22,23,24,25,26,27,28,29,30,31,32,33,34^ decreased,^35,36,37,38,39,40^ or no difference^23,26,28,29,31,41,42,43^ in brain activation responses to test stimuli.

The lack of consistency might be related to varying factors including the operationalization of adversity,^44,45,46^ which can include nutritional deprivation, sexual and physical abuse, war exposure, social isolation, and limited financial resources.^44,46,47^ Additionally, judgments about the amount of adversity vary, with some studies relying on frequency counts, some that separate levels of severity (eg, adversity vs trauma^44^), and others that make qualitative distinctions about different dimensions of adversity (eg, threat vs deprivation^47^).

Methodological factors may also be relevant. For many studies, this has included an overreliance on small samples, which may undermine reproducibility^48,49,50,51,52,53,54^ and inflate false-positive rates.^55^ However, even for large studies, the tested task domains vary and include emotion processing, threat processing, reward processing, memory processing, and executive control.^47,56,57^ Within the threat processing domain, some studies compare responses to fearful facial expressions with responses to neutral facial expressions (ie, emotional face task), whereas other studies measure responses during recall of traumatic experiences (ie, script-driven trauma-recall tasks). Finally, image acquisition and preprocessing protocols differ across studies,^51^ as do strategies for aggregating and drawing inferences from complex voxelwise analyses.^52^

Identifying trends in such a complex literature requires meta-analytic procedures that are robust to small samples and methodological variability. Two commonly used methods in neuroimaging meta-analyses are activation likelihood estimation (ALE) and multilevel kernel density analysis (MKDA). The results from ALE-based meta-analysis only generalize to peaks from the same studies because contrasts and studies are considered fixed effects, where interstudy variability in the number and location of peaks and each individual study’s statistical power remains unaccounted for.^55^ In comparison, in MKDA, contrasts and results are entered as random effects, where differences in statistical power and the number and location of peaks are taken into consideration, thereby facilitating the ability to generalize to new studies.^55,58,59^ Based on these features, MKDA analyses are considered more powerful for making inferences about both the consistency of activations across studies and the specificity of neural activity patterns associated with particular psychological processes.^58^ Based on the aforementioned issues, our study’s aim was to perform a systematic review and MKDA meta-analysis of human neuroimaging studies exploring the association of prior exposure to adversity with brain function, which included investigating whether some regions exhibit consistently altered activity as a function of (1) subtypes of adversity vs all forms of adversity, and (2) specific task domains vs multiple domains.

## Methods

**Figure 1.**
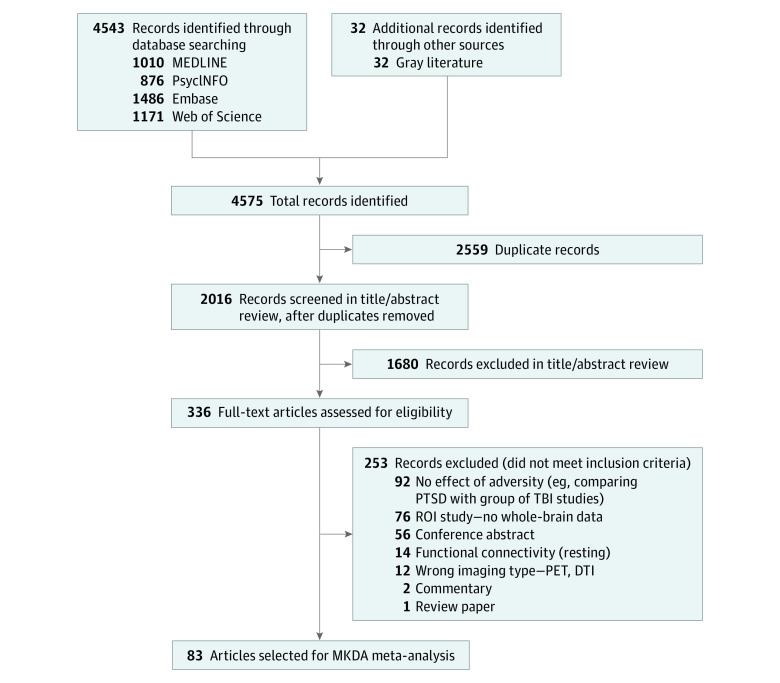
PRISMA Flowchart DTI indicates diffusion tensor imaging; MKDA, multilevel kernel density analysis; PET, positron emission tomography; PRISMA, preferred reporting items for systematic reviews and meta-analyses; PTSD, posttraumatic stress disorder; ROI, region of interest; TBI, traumatic brain injury.

This study follows the Preferred Reporting Items for Systematic Reviews and Meta-Analyses (PRISMA) reporting guideline (Figure 1). Literature searches were conducted within PsycINFO, Medline, EMBASE, and Web of Science from inception through May 4, 2022. We also searched the Brainmap database and gray literature for additional articles. The following search term combinations were used for each database: *trauma, posttraumatic stress disorder* (*PTSD*), *abuse, maltreatment, poverty, adversity,* or *stress*; and *functional magnetic resonance imaging *(*fMRI*) or *neuroimaging*; and *emotion*, *emotion regulation*, *memory, memory processing, inhibitory control, executive functioning*, *reward*, or *reward processing*. Articles were considered for review if these search terms were found in the title, abstract, section heading, table of contents, key concepts, original title, or tests and measures. Conference abstracts, books, reviews, meta-analyses, opinions, animal studies, articles not in English, and studies with fewer than 5 participants were excluded.

Our search yielded 2016 abstracts. Two independent reviewers (N.H.K. and S.S.) assessed abstracts for entry criteria. Conflicts were resolved by a third reviewer (M.V.F.). Of the 2016 abstracts, 336 articles met the criteria for full-text review. Full text articles were reviewed by 2 independent reviewers (N.H.K and M.V.F.). Data (x-axis, y-axis, and z-axis coordinates) were extracted by both reviewers, and a third reviewer (K.S.) resolved conflicts and errors in data extraction.

### Dimensional Model of Adversity

To test whether variability across studies was associated with differences in the operationalization of adversity,^44^ we stratified investigations on the basis of their definitions of adversity. One approach was to stratify studies according to whether they measured threat, defined as a challenge to physical integrity (eg, sexual and physical abuse); deprivation, defined as an absence of expected environmental inputs (eg, emotional neglect, institutionalization); or mixed adversity (ie, threat and deprivation combined).^47^ A second approach was to stratify studies by adversity severity to distinguish severe trauma from moderate adversities. Because there are no clear conceptual boundaries differentiating adversity from trauma,^44,60^ we used the *Diagnostic and Statistical Manual of Mental Disorders* (Fifth Edition [*DSM-5*]) Criterion A in trauma-related disorders to distinguish trauma from moderate adversities.

### Statistical Analysis

Peak x-axis (left-right), y-axis (posterior-anterior), and z-axis (inferior-superior) activation coordinates in Montreal Neurological Institute (MNI) or Talairach space were extracted from all included studies.^59^ We then stratified the reported coordinate activations according to the type of contrasts across the task domains (emotion processing, memory processing, inhibitory control, and reward processing) and participant groups (adversity exposure vs comparison group). The unit of analysis in MKDA is the sample size–weighted proportion of studies that report activation differences at a given voxel.^55,59^ The null hypothesis is that the activation distribution in the contrast indicator maps is distributed randomly and uniformly throughout the brain. Monte Carlo simulations with 10 000 iterations are used to compare the observed contrast map with the null distribution map. Peak coordinates of each map are separately convolved with a spherical kernel. A null distribution is simulated by permuting the activation locations and taking the weighted average iteratively on the basis of sample size; finally, a threshold (P<.05 familywise error rate [FWER]) adjusted for multiple comparisons is used to determine significance. Thus, significant results are areas where the consistency of activation across studies exceeds what would be expected by chance, where chance is a random even distribution across the whole brain.^55,59^ MKDA meta-analyses can, therefore, identify regions that are consistently or reliably activated in a set of studies.^55,59^ We performed our meta-analysis in MATLAB version R2021b using the MKDA toolbox.^61^ Data analysis was conducted from August to November 2022.

## Results

### All Neurocognitive Domains

**Figure 2.**
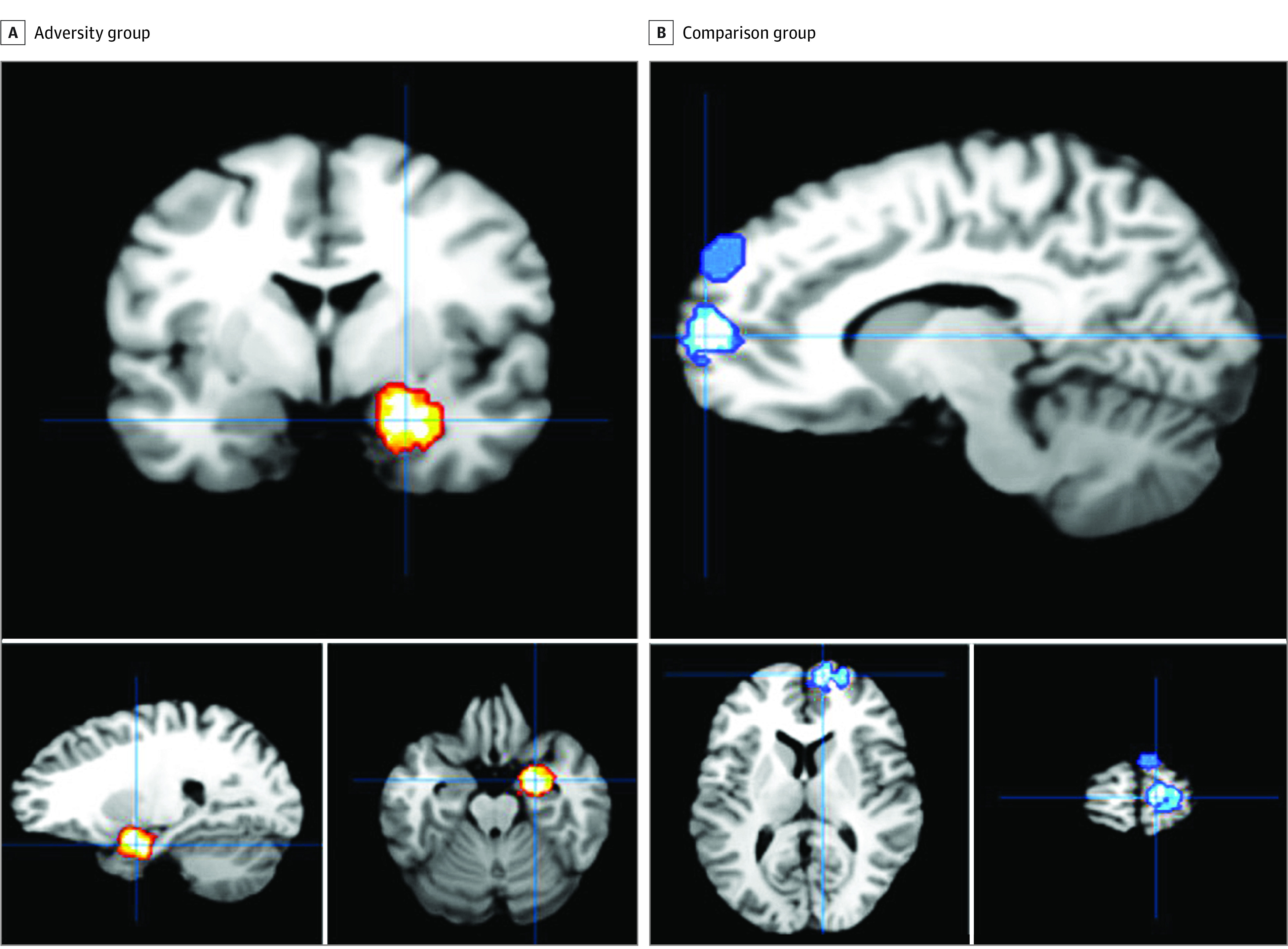
Coordinates Indicating Blood-Oxygen-Level-Dependent (BOLD) Responses for Adversity Group vs Comparison Group for All 4 Neurocognitive Domains Panel A shows collective images of studies that reported greater BOLD responses in the adversity group compared with the comparison group (67 studies; 523 coordinates) across all task domains (emotion processing, memory processing, inhibitory control, and reward processing). Right amygdala activation was consistently reported across studies in adversity groups compared with comparisons (familywise error rate corrected *P* < .001). Panel B shows collective images of studies that reported greater BOLD responses in the comparison group compared with the adversity group (47 studies; 278 coordinates). Greater prefrontal cortex activation was consistently reported across studies in comparison groups compared with adversity-exposed individuals (familywise error rate corrected *P* < .001).

A total of 83 studies^21,22,23,27,29,31,33,34,36,37,38,39,41,43,62,63,64,65,66,67,68,69,70,71,72,73,74,75,76,77,78,79,80,81,82,83,84,85,86,87,88,89,90,91,92,93,94,95,96,97,98,99,100,101,102,103,104,105,106,107,108,109,110,111,112,113,114,115,116,117,118,119,120,121,122,123,124,125,126,127,128,129,130,131,132^ were included with a pooled sample size of 5242 participants and 801 coordinates (eTable 1 and eTable 2 in Supplement 1). We entered 523 coordinates from the 67 studies^21,22,23,27,29,31,33,34,36,39,41,43,62,63,64,66,67,68,69,70,72,74,75,79,80,81,82,83,84,85,86,89,90,91,92,93,94,96,97,100,103,104,105,106,107,108,109,110,112,113,114,115,116,117,118,119,120,121,122,123,124,125,126,128,129,130,131^ that reported greater blood-oxygen-level-dependent (BOLD) responses in the adversity exposure group compared with the comparison group in at least 1 of the 4 neurocognitive domains (emotion processing, memory processing, inhibitory control, and reward processing) (Table). This analysis identified greater right amygdala responses in adversity exposure groups compared with comparisons (FWER corrected at *P* ≤ .001; x-axis = 22, y-axis = −4, z-axis = −17) (Figure 2A). Next, we entered 278 coordinates from 47 studies^29,33,37,38,39,43,62,64,65,68,69,70,71,73,75,76,77,78,80,83,86,87,88,91,93,94,95,96,99,100,101,104,105,106,108,109,111,114,116,122,123,124,125,127,129,130,132^ that reported lower BOLD responses in the adversity group relative to the comparison group. This yielded consistently lower PFC (medial frontal gyrus [Brodmann area 9]) BOLD responses in the adversity exposure group relative to the comparison group (FWER corrected at *P* < .001; x-axis = 10; y-axis = 60; z-axis = 10) (Figure 2B). Given the substantial variability in tasks used, we next separated studies by the 4 neurocognitive domains.

**Table.** Summary of All Analyses

Task domain^a^	Type of adversity^b^	Brain activation comparison^c^	No. of studies	No. of coordinates	Peak coordinates	Brain regions
All	All	Adversity > comparison	67^21,22,23,27,29,31,33,34,36,39,41,43,62,63,64,66,67,68,69,70,72,74,75,79,80,81,82,83,84,85,86,89,90,91,92,93,94,96,97,100,103,104,105,106,107,108,109,110,112,113,114,115,116,117,118,119,120,121,122,123,124,125,126,128,129,130,131^	523	x-axis = 22 y-axis = −4 z-axis = −17	Amygdala (right)
All	All	Comparison > adversity	47^29,33,37,38,39,43,62,64,65,68,69,70,71,73,75,76,77,78,80,83,86,87,88,91,93,94,95,96,99,100,101,104,105,106,108,109,111,114,116,122,123,124,125,127,129,130,132^	278	x-axis = 10 y-axis = 60 z-axis = 10	Prefrontal cortex (medial frontal gyrus [Brodmann Area 9])
Emotional processing	All	Adversity > comparison	45^21,22,23,27,29,31,34,36,39,43,63,66,67,68,72,73,74,75,80,81,82,84,86,89,90,91,94,96,97,100,103,104,105,106,108,114,115,116,117,120,124,125,126,128,129,131^	370	x-axis = 22 y-axis = −3 z-axis = −19	Amygdala
Emotional processing	All	Comparison > adversity	25^29,37,38,39,43,68,73,75,80,86,91,94,95,96,99,100,104,105,106,108,114,116,124,125,129^	140	x-axis = 22 y-axis = 61 z-axis = 13	Prefrontal cortex
Inhibitory control	All	Adversity > comparison	9^62,64,69,79,84,93,107,113,119^	52	x-axis = −26 y-axis = 19 z-axis = 13	Claustrum; anterior cingulate cortex; insula
Inhibitory control	All	Comparison > adversity	6^62,64,69,76,88,93^	14	None	None
Memory processing	All	Adversity > comparison	10^63,77,91,92,101,109,110,111,118,130^	33	None	None
Memory processing	All	Comparison > adversity	10^63,77,91,92,101,109,110,111,118,130^	64	None	None
Reward processing	All	Adversity > comparison	17^33,41,65,70,71,78,83,85,87,106,112,121,122,123,126,127,132^	122	None	None
Reward processing	All	Comparison > adversity	17^33,41,65,70,71,78,83,85,87,106,112,121,122,123,126,127,132^	65	None	None
All	Threat	Threat > comparison	32^21,27,31,34,39,63,64,68,72,80,81,82,89,90,92,96,97,100,105,106,107,108,110,115,116,121,123,124,125,126,129,131^	294	x-axis = 58 y-axis = −2 z-axis = 4	Superior temporal gyrus (right)
All	Threat	Comparison > threat	18^39,64,65,68,80,95,96,100,101,105,106,108,111,116,123,124,125,129^	133	x-axis = 61 y-axis = 22 z-axis = 13	Prefrontal cortex (medial frontal gyrus)
All	Mixed	Mixed > comparison	20^23,33,43,62,66,69,70,74,75,83,84,90,93,94,109,114,117,118,120,128^	114	x-axis = 14 y-axis = 50 z-axis = 39	Amygdala, precuneus, superior frontal gyrus
All	Mixed	Comparison > mixed	14^33,38,43,62,69,70,75,76,83,88,93,94,109,114^	87	None	None
All	Deprivation	Deprivation > comparison	8^22,29,36,37,41,113,131,132^	29	None	None
All	Deprivation	Comparison > deprivation	8^22,29,36,37,41,113,131,132^	15	None	None
All	Trauma	Trauma > comparison	56^21,22,27,29,31,33,34,36,39,41,43,62,63,64,66,68,69,72,74,75,80,81,82,83,84,89,90,92,93,94,96,97,100,104,105,106,107,108,109,110,113,114,115,116,117,118,120,121,123,124,125,126,128,129,130,131^	463	x-axis = 23 y-axis = −4 z-axis = 17	Amygdala (bilateral)
All	Trauma	Comparison > trauma	36^29,33,37,38,39,43,62,64,65,68,69,73,75,76,80,83,88,93,94,95,96,100,101,104,105,106,108,109,111,114,116,123,124,125,129,130^	232	x-axis = 15 y-axis = 62 z-axis = 9	Prefrontal cortex (medial frontal gyrus)
All	Moderate adversity	Moderate adversity > comparison	18^23,67,70,71,77,78,79,85,86,87,91,99,103,112,119,122,127,132^	61	None	None
All	Moderate adversity	Comparison > moderate adversity	18^23,67,70,71,77,78,79,85,86,87,91,99,103,112,119,122,127,132^	45	None	None
All	All	PTSD > comparisons	20^31,43,63,68,72,80,81,82,90,92,97,106,107,116,123,124,126,129,130,131^	219	x-axis = −30 y-axis = −17 z-axis = −18	Amygdala (left)
All	All	Comparisons > PTSD	13^38,43,65,68,80,101,106,111,116,123,124,129,130^	127	x-axis = −36 y-axis = 5 z-axis = −5	Hippocampus, orbitofrontal cortex, insula, striatum
All	All	Adults: adversity > comparison	34^23,66,67,68,70,72,74,79,80,84,85,86,89,90,91,92,104,106,110,114,115,116,117,118,119,120,121,123,124,125,126,129,130,131^	282	x-axis = 24 y-axis = −4 z-axis = −17	Amygdala (right)
All	All	Adults: comparison > adversity	23^65,68,70,76,77,80,87,88,91,94,99,101,104,106,111,114,116,123,124,125,127,129,130^	156	x-axis = 29 y-axis = 60 z-axis = 10	Prefrontal cortex (middle frontal gyrus [Brodmann Area 10])
All 4 domains	All types	Adolescents: adversity > comparison	15^21,27,31,64,69,81,82,83,96,97,100,105,106,107,113,122^	121	None	None
All 4 domains	All types	Adolescents: comparison > adversity	11^64,69,71,78,83,95,96,100,105,122,132^	121	None	None
All 4 domains	All types	Children: adversity > comparison	16^22,29,33,34,36,39,41,43,62,75,93,103,108,109,112,128^	115	None	None
All 4 domains	All types	Children: comparison > adversity	11^29,33,37,38,39,43,62,73,75,108,109^	66	None	None

^a^ Domains included emotion processing, memory processing, inhibitory control, and reward processing.

^b^ Adversity types included threat (challenge to physical integrity), deprivation (absence of expected environmental inputs), or mixed (threat and deprivation combined).

^c^ For comparisons, > indicates greater blood oxygen level dependent (BOLD) responses.

### Emotional Processing

Restricting the analysis to the 50 studies^21,22,23,27,29,31,34,36,37,38,39,43,63,66,67,68,72,73,74,75,80,81,82,84,86,89,90,91,94,95,96,97,99,100,103,104,105,106,108,114,115,116,117,120,124,125,126,128,129,131^ (3413 participants) that administered emotion processing tasks yielded greater amygdala reactivity (45 studies^21,22,23,27,29,31,34,36,39,43,63,66,67,68,72,73,74,75,80,81,82,84,86,89,90,91,94,96,97,100,103,104,105,106,108,114,115,116,117,120,124,125,126,128,129,131^; 370 coordinates; FWER-corrected *P* < .05; x = 22, y = −3, z = −19) (eFigure 1 in Supplement 2) and lower PFC (superior frontal gyrus [Brodmann area 10]) reactivity in the adversity exposure group compared with controls (25 studies^29,37,38,39,43,68,73,75,80,86,91,94,95,96,99,100,104,105,106,108,114,116,124,125,129^; 140 coordinates; FWER-corrected *P* < .05; x-axis = 22; y-axis = 61; z-axis = 13) (eFigure 2 in Supplement 2).

### Inhibitory Control, Memory Processing, and Reward Processing

When analyzing the 11 studies^62,64,69,76,79,84,88,93,107,113,119^ (425 participants) that administered inhibitory control tasks, greater activity was found in the claustrum, anterior cingulate cortex (ACC), and insula in the adversity exposure group compared with controls (9 studies^62,64,69,79,84,93,107,113,119^; 52 coordinates; FWER-corrected *P* < .05; axis = −26, y-axis = 19; z-axis = 13) (eFigure 3 in Supplement 2). No group differences were identified when looking at studies that reported lower BOLD responses (6 studies^62,64,69,76,88,93^; 14 coordinates). There were no statistically significant group differences in the 10 studies^63,77,91,92,101,109,110,111,118,130^ that used memory tasks (440 participants; 97 coordinates [33 coordinates for adversity groups showing heightened BOLD responses in the adversity group compared with controls; 64 coordinates showing greater BOLD responses in the control groups compared with the adversity-exposed groups]) or reward processing tasks (17 studies^33,41,65,70,71,78,83,85,87,106,112,121,122,123,126,127,132^; 1265 participants; 187 coordinates [122 coordinates reporting greater BOLD responses in adversity-exposed participants relative to controls; 65 coordinates showing greater BOLD responses in the control groups compared with the adversity-exposed groups]).

### Threat

**Figure 3.**
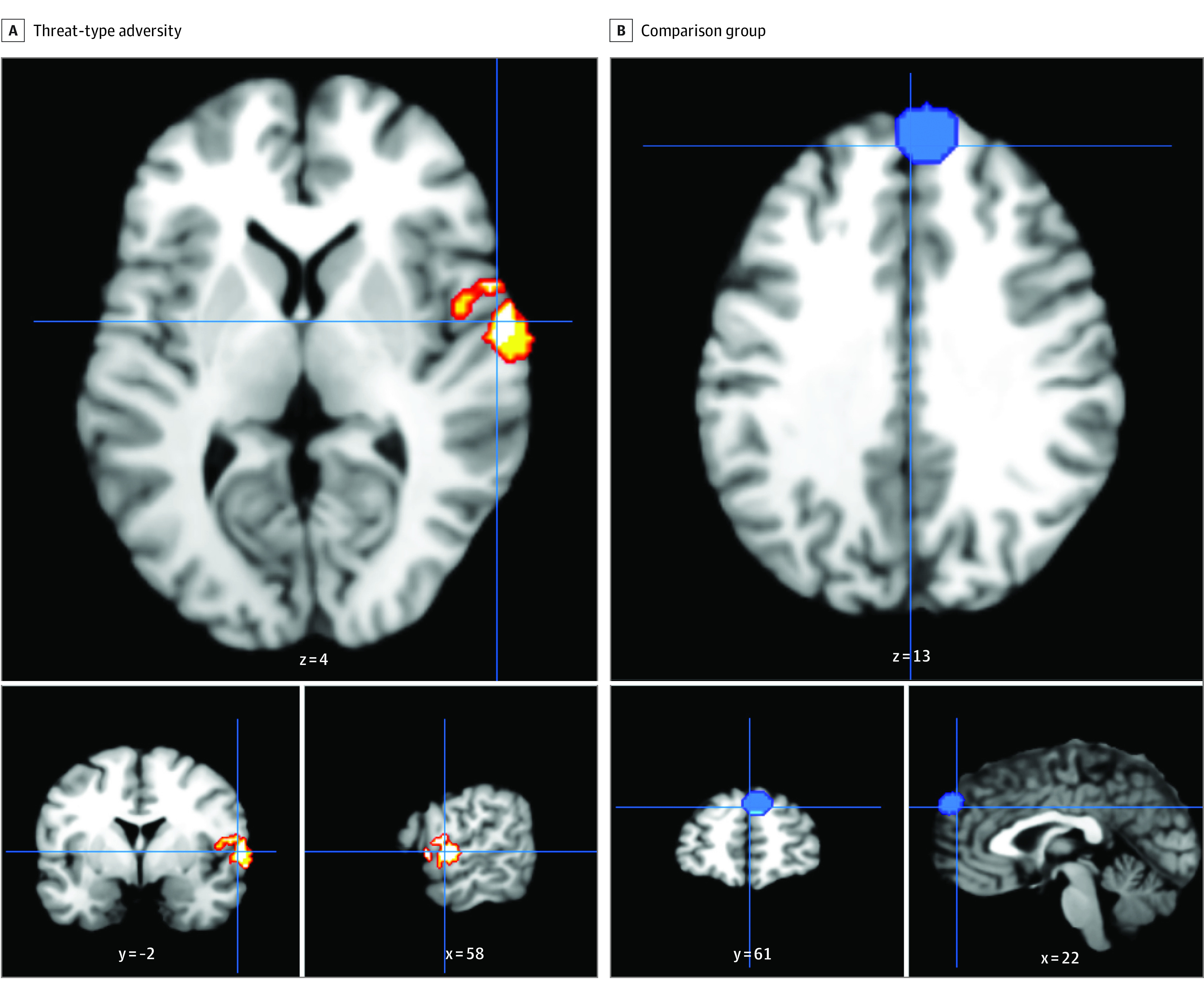
Coordinates Indicating Blood-Oxygen-Level-Dependent (BOLD) Responses for Threat-Type Adversity Group vs Comparison Group Panel A shows collective images of studies that reported greater BOLD responses in threat-exposed adversity groups compared with comparison groups (32 studies; 294 coordinates). Greater superior temporal gyrus activation was consistently reported across studies in individuals exposed to threat types of adversity as compared with controls (familywise error rate corrected *P* < .05). Panel B shows collective images of studies that reported greater BOLD responses in the comparison group compared with threat-exposed adversity group (18 studies; 133 coordinates). Lower prefrontal cortex (medial frontal gyrus) activity was seen in participants exposed to threat compared with comparisons (familywise error rate corrected *P* < .05).

In the 36 studies^21,27,31,34,39,63,64,65,68,72,80,81,82,89,90,92,95,96,97,100,101,105,106,107,108,110,111,115,116,121,123,124,125,126,129,131^ (1899 participants; 427 coordinates) in which threat types of adversity were associated with brain function in any of the 4 task domains, we found greater BOLD responses in the superior temporal gyrus (32 studies^21,27,31,34,39,63,64,68,72,80,81,82,89,90,92,96,97,100,105,106,107,108,110,115,116,121,123,124,125,126,129,131^; 294 coordinates; FWER-corrected *P* < .05; x-axis = 58, y-axis = −2; z-axis = 4) (Figure 3A) and lower PFC activity (medial frontal gyrus) in participants exposed to threat compared with controls (18 studies^39,64,65,68,80,95,96,100,101,105,106,108,111,116,123,124,125,129^; 133 coordinates; FWER-corrected *P* < .05; x-axis = 61; y-axis = 22; z-axis = 13) (Figure 3B). These findings were seen across task domains.

### Mixed Adversity

When we analyzed the 23 studies^23,33,38,43,62,66,69,70,74,75,76,83,84,88,90,93,94,109,114,117,118,120,128^ (1636 participants; 201 coordinates) using mixed types of adversity to create groups, greater activity across all 4 task domains was found in the right amygdala, precuneus, and superior frontal gyrus in individuals exposed to mixed-type adversities compared with the comparison groups (20 studies^23,33,43,62,66,69,70,74,75,83,84,90,93,94,109,114,117,118,120,128^; 114 coordinates; FWER-corrected *P* < .05; x-axis = 14, y-axis = 50; z-axis = 39) (eFigure 4 in Supplement 2). No group differences were identified when looking at studies that reported lower BOLD responses in individuals exposed to mixed-type adversities compared with comparisons (14 studies^33,38,43,62,69,70,75,76,83,88,93,94,109,114^; 87 coordinates).

### Deprivation

The 8 studies^22,29,36,37,41,113,131,132^ that reported on deprivation-type adversities alone did not yield significant results. Given the small number of studies, it remains difficult to draw confident conclusions.

### Adversity: Severity Model

To determine whether traumatic vs moderately adverse events yield different activation profiles, we stratified studies on the basis of whether the adversity measured in each study met *DSM-5 *criterion A for trauma-related disorders. Studies that used questionnaires that appeared to meet this criterion were coded as trauma type adversities (65 studies^21,22,27,29,31,33,34,36,37,38,39,41,43,62,63,64,65,66,68,69,72,73,74,75,76,80,81,82,83,84,88,89,90,92,93,94,95,96,97,100,101,104,105,106,107,108,109,110,111,113,114,115,116,117,118,120,121,123,124,125,126,128,129,130,131^; 4003 participants; 695 coordinates), whereas those that did not meet criteria were coded as moderate adversities (18 studies^23,67,70,71,77,78,79,85,86,87,91,99,103,112,119,122,127,132^; 1239 participants; 106 coordinates). This analysis yielded significantly greater bilateral amygdala activation (56 studies^21,22,27,29,31,33,34,36,39,41,43,62,63,64,66,68,69,72,74,75,80,81,82,83,84,89,90,92,93,94,96,97,100,104,105,106,107,108,109,110,113,114,115,116,117,118,120,121,123,124,125,126,128,129,130,131^; 463 coordinates; FWER-corrected *P* < .05; x-axis = 23; y-axis = −4; z-axis = 17) (eFigure 5 in Supplement 2) and significantly lower PFC (medial frontal gyrus [Brodmann area 10]), ACC, and striatal responses in participants with a history of trauma-type adversities compared with the comparison group (36 studies^29,33,37,38,39,43,62,64,65,68,69,73,75,76,80,83,88,93,94,95,96,100,101,104,105,106,108,109,111,114,116,123,124,125,129,130^; 232 coordinates; FWER corrected *P* < .05; x = 15; y = 62; z = 9) (eFigure 6 in Supplement 2). In contrast, our analysis of the association of moderate adversities with brain function did not yield any significant results (18 studies^23,67,70,71,77,78,79,85,86,87,91,99,103,112,119,122,127,132^; 61 coordinates showing greater BOLD responses in the groups exposed to moderate adversity as compared with controls; 45 coordinates showing greater BOLD responses in control group participants as compared with the moderate adversity groups). These results were obtained across task domains.

### Trauma-Related Psychopathology

Because experiences of adversity and trauma are associated with diagnoses of mood and trauma-related psychopathologies, we conducted MKDA analysis for studies that reported findings in participants with a confirmed diagnosis of PTSD. These analyses yielded significantly greater left amygdala activation (20 studies^31,43,63,68,72,80,81,82,90,92,97,106,107,116,123,124,126,129,130,131^; 219 coordinates; FWER corrected *P* < .05; x-axis = −30; y-axis = −17 [Brodmann area 13]; z-axis = −18) (eFigure 7 in Supplement 2) and significantly lower hippocampal, orbitofrontal cortex, striatal, and insula activity in individuals with PTSD compared with the comparison group (13 studies^38,43,65,68,80,101,106,111,116,123,124,129,130^; 127 coordinates; FWER-corrected *P* < .05; x-axis = −36; y-axis = 5; z-axis = −5) (eFigure 8 in Supplement 2). These effects were also seen across task domains.

### Developmental Considerations

Some studies collected data from adults, whereas others tested adolescents or children. We therefore stratified studies according to whether participants were adults (aged ≥18 years), adolescents (aged 13-17 years), or children (aged ≤12 years).

### Adults

We entered 282 coordinates from 34 studies^23,66,67,68,70,72,74,79,80,84,85,86,89,90,91,92,104,106,110,114,115,116,117,118,119,120,121,123,124,125,126,129,130,131^ that reported greater BOLD responses in adversity exposure compared with controls in adulthood (2382 participants; mean [SD] age, 32.7 [6.4] years). The analysis yielded significantly greater right amygdala activations (FWER-corrected *P* < .001; x-axis = 24; y-axis = −4, z-axis = −17) (eFigure 9 in Supplement 2) and significantly lower activations in the middle frontal gyrus (Brodmann area 10; 1074 participants; mean [SD] age, 31.2 [7.1] years; 23 studies^65,68,70,76,77,80,87,88,91,94,99,101,104,106,111,114,116,123,124,125,127,129,130^; 156 coordinates; FWER-corrected *P* < .001; x-axis = 29; y-axis = 60; z-axis = 10) in adversity-exposed adults compared with controls (eFigure 10 in Supplement 2).

### Adolescents

Next, we analyzed data from 15 studies^21,27,31,64,69,81,82,83,96,97,100,105,106,107,113,122^ in adolescents (685 participants; mean [SD] age, 15.3 [1.5] years; 121 coordinates) that reported greater BOLD responses in brain regions associated with adversity exposure. This analysis did not find significantly reliable clusters of activation across the studies. Likewise, no group differences were identified when looking at 11 studies^64,69,71,78,83,95,96,100,105,122,132^ that reported lower BOLD responses in adolescents exposed to adversity compared with controls (686 participants; mean [SD] age, 14.9 [1.3] years; 121 coordinates).

### Children

Sixteen studies^22,29,33,34,36,39,41,43,62,75,93,103,108,109,112,128^ (908 participants; mean [SD] age, 11.7 [1.5] years; 115 coordinates) reported greater BOLD responses in children exposed to prior adversity compared with controls, but the meta-analysis yielded no significant clusters of activation. Eleven studies^29,33,37,38,39,43,62,73,75,108,109^ reported blunted BOLD responses in adversity-exposed children compared with comparisons; again, however, this yielded no significant results (725 participants; mean [SD] age, 11.0 [0.95] years; 66 coordinates).

## Discussion

The primary results of this meta-analysis provide clear evidence that a history of severe adversity is associated with long-lasting increases in adult amygdala responses and decreases in PFC responses to diverse psychological challenges. These outcomes were obtained by using MKDA, allowing us to overcome some of the limitations of ALE-based analyses and to probe the literature in a manner that is both more comprehensive (83 studies) and more generalizable to new research. The core results may be due to a diminished ability of the PFC to downregulate amygdala reactivity via white matter fiber tracts connecting the regions.^133,134,135^

The amygdala is critically involved in threat detection and generating defensive behaviors.^6,133^ Indeed, amygdala hyperactivity has been implicated as a biomarker for PTSD diagnosis and is associated with symptom severity.^134,135,136,137^ Consistent with this finding, 2 previous ALE meta-analyses^138,139^ found that amygdala hyperactivity distinguishes PTSD from both healthy controls and patients with depression. Likewise, multiple narrative reviews^62,134,137,140,141^ concluded that people with PTSD exhibit heightened amygdala reactivity and PFC hyporesponsiveness during emotion processing tasks.

When exploring domain-specific outcomes, we replicated the findings of heightened amygdala reactivity and blunted PFC reactivity when analyzing responses to emotional processing tasks alone. In comparison, heightened amygdala and blunted PFC reactivity was observed across task domains when comparing people with histories of severe vs more moderate adversities and PTSD vs the comparison groups. The latter 2 analyses also identified evidence of decreased striatal reactivity, potentially reflecting a diminished ability to translate motivational states into action.^142,143^

In various subanalyses with progressively smaller sample sizes, the results became more variable; however, some of the results are intriguing. In studies that measured responses to inhibitory control tasks, adversity exposure was associated with heightened insula and ACC responses. Greater ACC activity in adversity-exposed individuals is consistent with reports of heightened ACC activity in severe abuse and PTSD^63,144,145,146^ and could reflect the region’s contribution to processing ambiguous information. The insula, in comparison, integrates internal and external information to create subjective awareness and is implicated in cognitive emotional evaluation.^147,148,149,150^ The finding of insula activation in adversity-exposed individuals is consistent with previous literature,^62,64,151,152,153,154,155,156^ but contrary to numerous individual reports, our analyses yielded this effect only in the inhibitory control tasks, which could reflect the insula’s role in the salience network and its recruitment during cognitively demanding tasks.^157,158^

Threat type adversities were associated with heightened right superior temporal gyrus activation, a region implicated in nonverbal sound discrimination, recognition, and comprehension. As seen in our pooled analysis, blunted PFC activity was found in threat-exposed individuals compared with controls, and heightened amygdala activity was observed in those exposed to mixed-type adversities. Mixed-type adversities were also associated with heightened activity in the precuneus, part of the default mode network with the posterior region being involved in episodic memory retrieval.^159,160^

We next conducted subanalyses for traumatic vs moderate adversities. This analysis recapitulated our general findings, showing increased amygdala activation and reduced PFC activation in trauma-exposed individuals. In contrast, significant activations were not found when analyzing prior moderate adversities. This difference between traumas and moderate adversity is compelling, although moderate adversity is a broad conceptualization and different authors have defined it as poverty, minority stress, and sexual orientation identity stress.

Finally, to examine the association of PTSD with brain function, we stratified studies on the basis of diagnosis. We found significantly greater left amygdala activity in participants with PTSD, compared with those with mood disorders and healthy controls. This finding contrasts with previous studies that implicated right amygdala hyperreactivity as a PTSD biomarker,^137^ as well as our omnibus analysis that identified increased right amygdala activity in individuals exposed to prior adversity and bilateral amygdala effects in those who had experienced trauma compared with moderate adversity. These discrepant findings may reflect functional differences between the left and right amygdala^161,162,163^ or continued statistical power issues. Finally, individuals with PTSD compared with controls also exhibited reduced hippocampal, orbitofrontal cortex, insula, and striatal activity. These findings are consistent with previous reports of diminished functional integrity of the hippocampus,^65,137,164^ striatum,^65,165^ and insula^138,166,167,168,169,170^ in individuals with PTSD.

### Limitations

The present study has several limitations to consider. First, most of the literature analyzing the association of deprivation with brain function reports region-of-interest data instead of whole-brain coordinates, thereby precluding their inclusion in the current analysis. Second, we categorized adversity type according to the *DSM-5* Criterion A type traumas, but we cannot be certain that these traumas were present or absent. In addition, adversity exposure is also associated with substance use, which might be a mediating variable; because studies entered in the meta-analysis did not control for substance use, this requires further research.

## Conclusions

Previous evidence has suggested that adversity exposure is associated with alterations within limbic and frontal cortical structures. However, single neuroimaging studies have low reliability and reproducibility. Our results from the present MKDA meta-analysis of fMRI studies provide evidence that prior adversity exposure is associated with exaggerated adult reactivity in brain regions that detect threat and reductions in regions that contribute to top-down cognitive-emotional control. Together, these effects might provide a mechanism by which prior adversity leads to long-lasting susceptibility to mental health problems.

## Notes

Supplement 1. **eTable 1.** All Studies **eTable 2.** Exclusions and Additions

Supplement 2. **eFigure 1.** Emotional Processing Tasks: Adversity > Comparison **eFigure 2.** Emotional Processing Tasks: Comparison > Adversity **eFigure 3.** Inhibitory Control Tasks: Adversity > Comparison **eFigure 4.** Mixed-Type Adversity: Mixed Adversity > Comparison **eFigure 5.** Severity of Adversity Trauma-Type Adversity > Comparison **eFigure 6.** Severity of Adversity Comparison > Trauma-Type Adversity **eFigure 7.** PTSD Diagnosis PTSD > Comparison **eFigure 8.** PTSD Diagnosis Comparison > PTSD **eFigure 9.** Adults Adversity > Comparison **eFigure 10.** Adults Comparison > Adversity

Supplement 3.
Data Sharing Statement
