# Supplementary material for: Adverse Life Experiences and Brain Function: A Meta-Analysis of Functional Magnetic Resonance Imaging Findings
Source: JAMA Netw Open. 2023 Nov 1;6(11):e2340018. doi: 10.1001/jamanetworkopen.2023.40018 (PMC10620621; doi:10.1001/jamanetworkopen.2023.40018)
Supplement: Supplement 2. — eFigure 1. Emotional Processing Tasks: Adversity > Comparison eFigure 2. Emotional Processing Tasks: Comparison > Adversity eFigure 3. Inhibitory Control Tasks: Adversity > Comparison eFigure 4. Mixed-Type Adversity: Mixed Adversity > Comparison eFigure 5. Severity of Adversity Trauma-Type Adversity > Comparison eFigure 6. Severity of Adversity Comparison > Trauma-Type Adversity eFigure 7. PTSD Diagnosis PTSD > Comparison eFigure 8. PTSD Diagnosis Comparison > PTSD eFigure 9. Adults Adversity > Comparison eFigure 10. Adults Comparison > Adversity [file jamanetwopen-e2340018-s002.pdf]

Supplemental Online Content

Hosseini-Kamkar N, Varvani Farahani, M, Nikolic M, et al. Adverse life experiences and brain function: a meta-analysis of functional magnetic resonance imaging findings. *JAMA Netw Open.* 2023;6(10):e2340018. doi:10.1001/jamanetworkopen.2023.40018

eFigure 1. Emotional Processing Tasks: Adversity > Comparison
eFigure 2. Emotional Processing Tasks: Comparison > Adversity
eFigure 3. Inhibitory Control Tasks: Adversity > Comparison
eFigure 4. Mixed-Type Adversity: Mixed Adversity > Comparison
eFigure 5. Severity of Adversity Trauma-Type Adversity > Comparison
eFigure 6. Severity of Adversity Comparison > Trauma-Type Adversity
eFigure 7. PTSD Diagnosis PTSD > Comparison
eFigure 8. PTSD Diagnosis Comparison > PTSD
eFigure 9. Adults Adversity > Comparison
eFigure 10. Adults Comparison > Adversity

This supplemental material has been provided by the authors to give readers additional information about their work.

© 2023 Hosseini-Kamkar N et al. *JAMA Network Open.*

eFigure 1: Emotional processing tasks
Adversity > Comparison

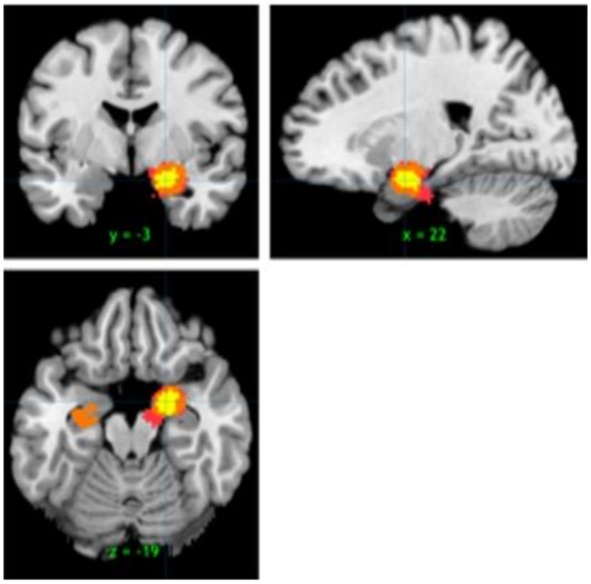

eFigure 2: Emotional processing tasks:
Comparison > Adversity

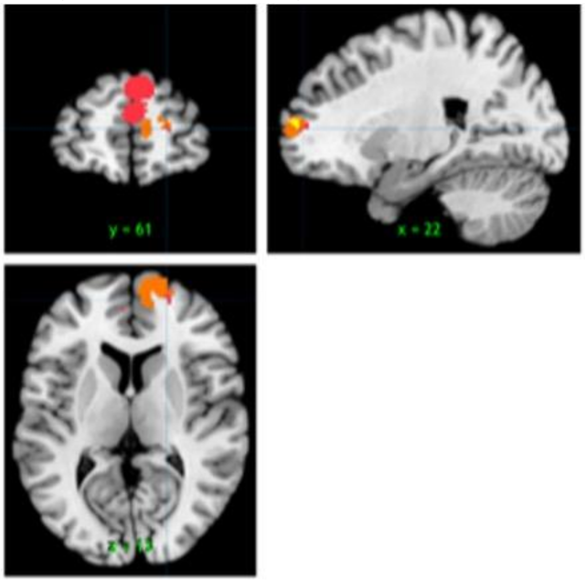

© 2023 Hosseini-Kamkar N et al. *JAMA Network Open.*

eFigure 3: Inhibitory Control tasks:
Adversity > Comparison

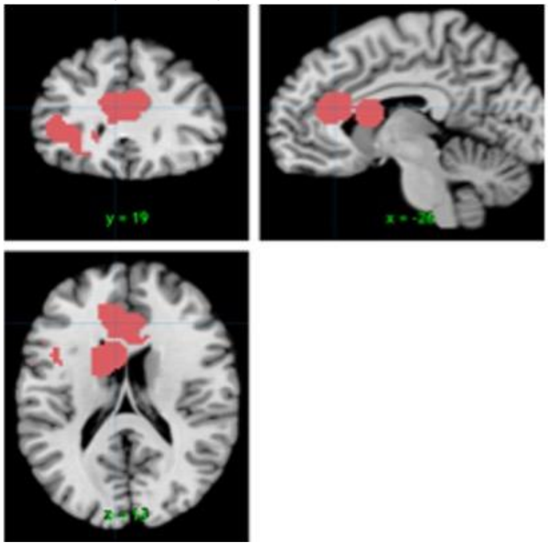

eFigure 4: Mixed-Type Adversity:
Mixed Adversity > Comparison

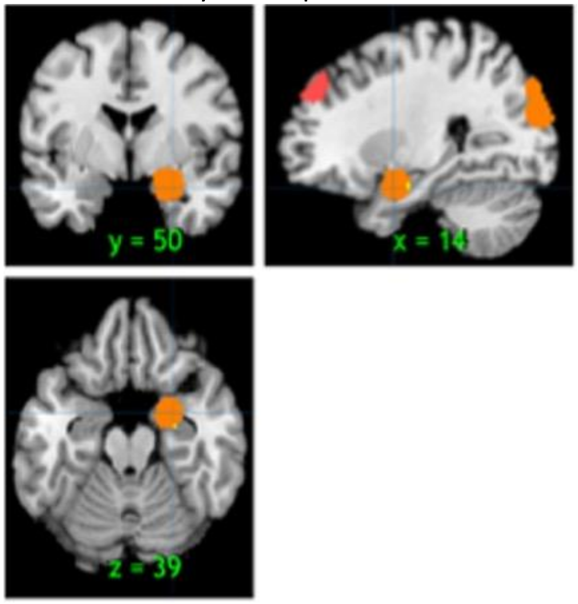

© 2023 Hosseini-Kamkar N et al. *JAMA Network Open.*

eFigure 5: Severity of Adversity
Trauma-Type Adversity > Comparison

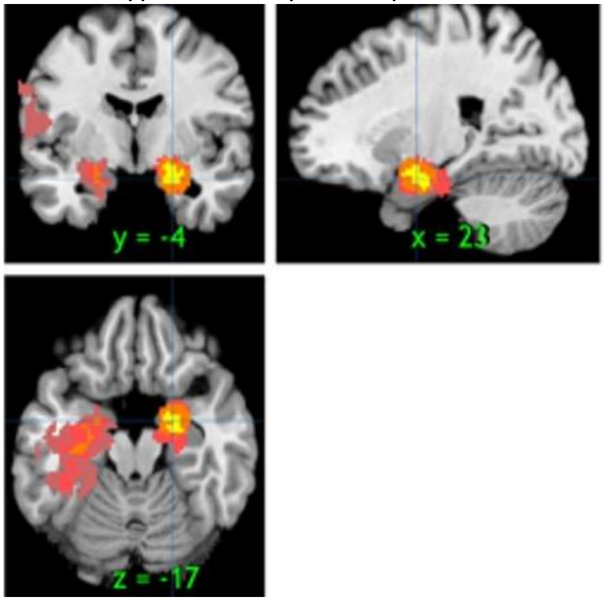

eFigure 6: Severity of Adversity
Comparison > Trauma-Type Adversity

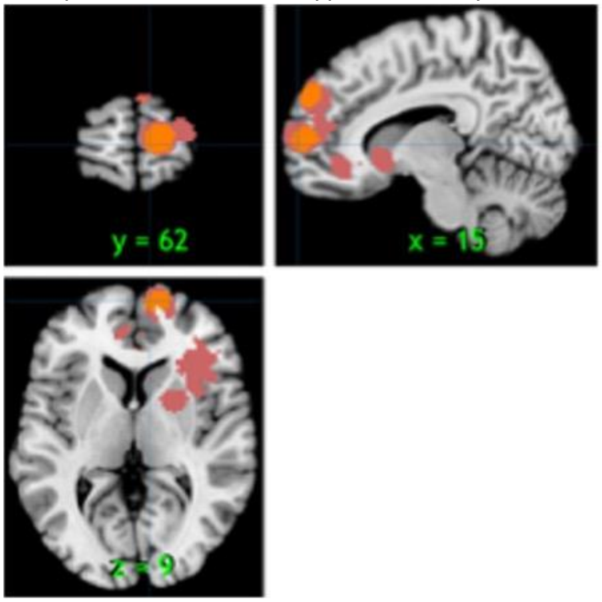

© 2023 Hosseini-Kamkar N et al. *JAMA Network Open.*

eFigure 7: PTSD Diagnosis
PTSD > Comparison

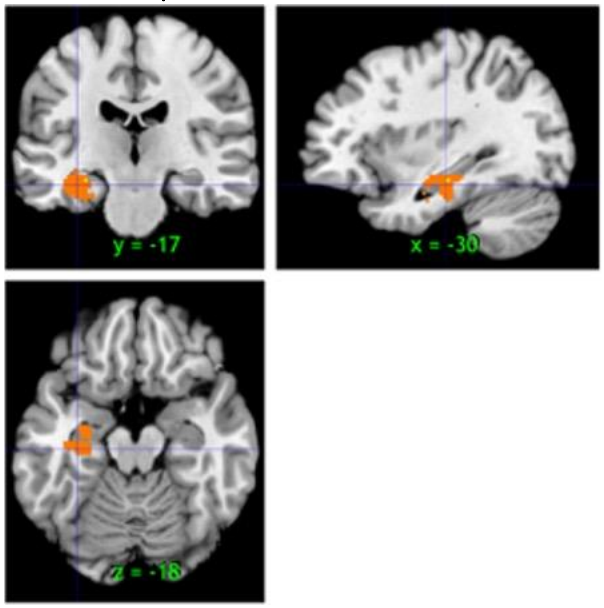

eFigure 8: PTSD Diagnosis
Comparison > PTSD

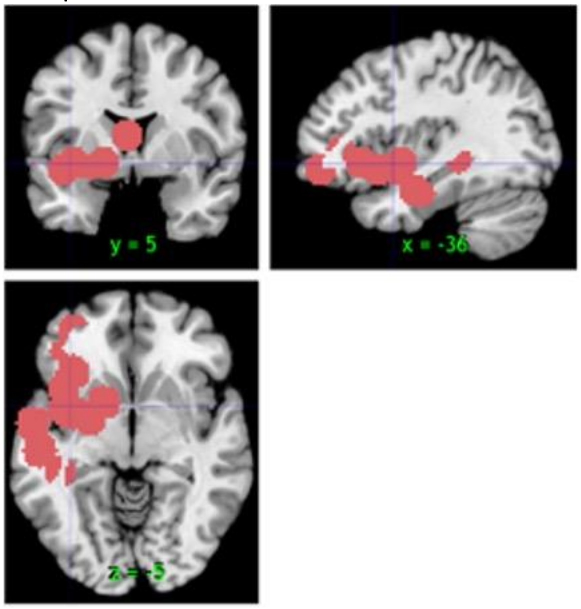

© 2023 Hosseini-Kamkar N et al. *JAMA Network Open.*

eFigure 9: Adults
Adversity > Comparison

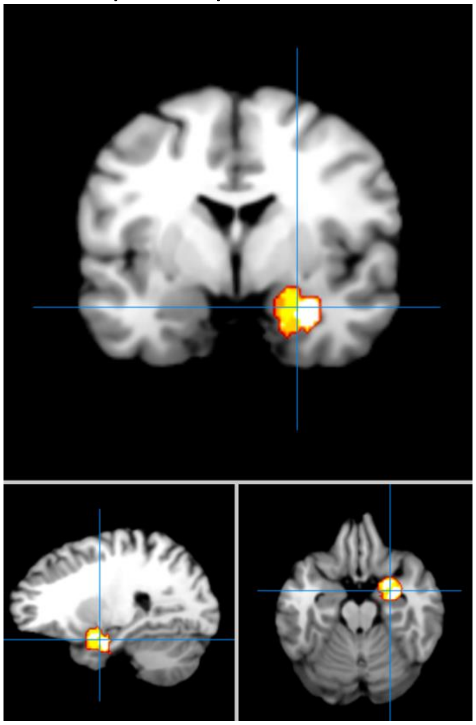

eFigure 10: Adults
Comparison > Adversity

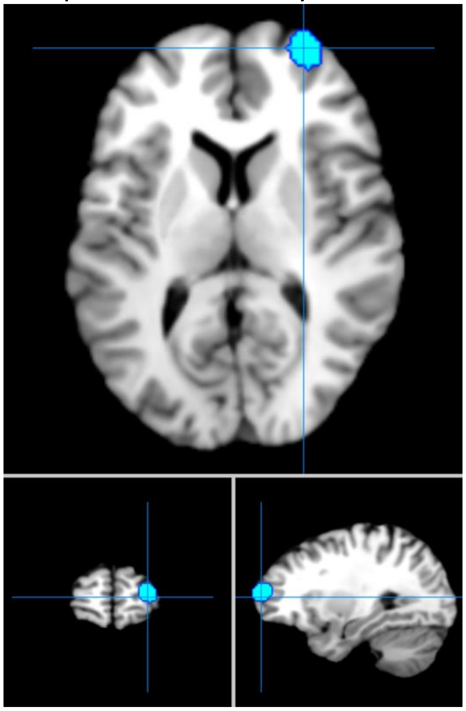

© 2023 Hosseini-Kamkar N et al. *JAMA Network Open.*
