# Supplementary material for: Adverse Life Experiences and Brain Function: A Meta-Analysis of Functional Magnetic Resonance Imaging Findings
Source: JAMA Netw Open. 2023 Nov 1;6(11):e2340018. doi: 10.1001/jamanetworkopen.2023.40018 (PMC10620621; doi:10.1001/jamanetworkopen.2023.40018)
Supplement: Supplement 3. — Data Sharing Statement [file jamanetwopen-e2340018-s003.pdf]

Data Sharing Statement

Hosseini-Kamkar. Adverse Life Experiences and Brain Function. *JAMA Netw Open*. Published November 01, 2023. doi:10.1001/jamanetworkopen.2023.40018

Data
Data available: Yes
Data types: Data (not involving human participants)
How to access data: niki.hosseini-kamkar@theroyal.ca Data are available online in Excel Sheet
When available: With publication

Supporting Documents
Document types: None

Additional Information
Who can access the data: Anyone requesting the data
Types of analyses: For any purposes
Mechanisms of data availability: With investigator support
